# Expert opinion on NSCLC small specimen biomarker testing — Part 1: Tissue collection and management

**DOI:** 10.1007/s00428-022-03343-2

**Published:** 2022-07-20

**Authors:** Frédérique Penault-Llorca, Keith M. Kerr, Pilar Garrido, Erik Thunnissen, Elisabeth Dequeker, Nicola Normanno, Simon J. Patton, Jenni Fairley, Joshua Kapp, Daniëlle de Ridder, Aleš Ryška, Holger Moch

**Affiliations:** 1 University Clermont Auvergne, INSERM U1240, Centre Jean Perrin, Clermont-Ferrand, France; 2 Department of Pathology, Aberdeen University Medical School and Aberdeen Royal Infirmary, Aberdeen, UK; 3 Medical Oncology Department, Hospital Universitario Ramón Y Cajal, University of Alcalá, Madrid, Spain; 4 Amsterdam University Medical Center, VU Medical Center, Amsterdam, the Netherlands; 5 Department of Public Health, Biomedical Quality Assurance Research Unit, Campus Gasthuisberg, University Leuven, Leuven, Belgium; 6 Cell Biology and Biotherapy Unit, Istituto Nazionale Tumori “Fondazione Giovanni Pascale” IRCCS, Naples, Italy; 7 EMQN CIC, Manchester, UK; 8 GenQA, Edinburgh, UK; 9 Amgen (Europe) GmbH, Rotkreuz, Switzerland; 10 Amgen BV, Breda, the Netherlands; 11 Department of Pathology, Charles University Medical Faculty Hospital, Hradec Králové, Czech Republic; 12 Department of Pathology and Molecular Pathology, University Hospital Zurich and University of Zurich, Zurich, Switzerland

**Keywords:** Best practice, Biopsy, Cytological techniques, Histology, Molecular diagnostics, Non-small cell lung carcinoma

## Abstract

Biomarker testing is crucial for treatment selection in advanced non-small cell lung cancer (NSCLC). However, the quantity of available tissue often presents a key constraint for patients with advanced disease, where minimally invasive tissue biopsy typically returns small samples. In Part 1 of this two-part series, we summarise evidence-based recommendations relating to small sample processing for patients with NSCLC. Generally, tissue biopsy techniques that deliver the greatest quantity and quality of tissue with the least risk to the patient should be selected. Rapid on-site evaluation can help to ensure sufficient sample quality and quantity. Sample processing should be managed according to biomarker testing requirements, because tissue fixation methodology influences downstream nucleic acid, protein and morphological analyses. Accordingly, 10% neutral buffered formalin is recommended as an appropriate fixative, and the duration of fixation is recommended not to exceed 24–48 h. Tissue sparing techniques, including the ‘one biopsy per block’ approach and small sample cutting protocols, can help preserve tissue. Cytological material (formalin-fixed paraffin-embedded [FFPE] cytology blocks and non-FFPE samples such as smears and touch preparations) can be an excellent source of nucleic acid, providing either primary or supplementary patient material to complete morphological and molecular diagnoses. Considerations on biomarker testing, reporting and quality assessment are discussed in Part 2.

## Introduction

The growing armamentarium of biomarker-directed therapies available for the treatment of advanced non-small cell lung cancer (NSCLC) [1] has improved outcomes for patients receiving targeted therapy versus cytotoxic therapy [2–5]. Thus, biomarker testing is essential to provide patients with the best standard of care.

Obtaining sufficient biopsy material to complete both morphological and molecular diagnostics is a challenge unique to advanced NSCLC due to two key factors. Firstly, the number of actionable molecular targets in NSCLC is higher than in other solid tumours. Secondly, the majority (~ 70%) of patients with NSCLC present with advanced disease where curative surgery is no longer feasible [6, 7]. Minimally invasive tissue biopsy techniques, such as core-needle biopsy (CNB) and fine-needle aspiration (FNA), are performed in such cases, but they yield smaller samples than surgical resection [7, 8]; therefore, it is important to preserve and optimally utilise these tissues [7, 8]. Small samples with limited tumour cell content (< 30%) may permit morphological classification; however, the quantity of tumour tissue is not always sufficient for biomarker testing [9, 10] and rebiopsies are not always possible [10–13]. Best practice guidelines aim to conserve tissue and enable a complete molecular diagnosis, so that eligible patients may benefit from targeted therapy.

The first article of this two-part review summarises evidence-based recommendations relating to the processing of small biopsy samples in NSCLC. Where no guidelines exist, collective recommendations are reported based on the experience of the author group. The second article [14] provides recommendations for biomarker testing, quality assessment and reporting.

## Biopsy techniques and sample acquisition methods

The selection of the biopsy technique is informed by many factors including the patient’s clinical condition, disease stage and site, availability of equipment and expertise [8, 15, 16]. While biopsy techniques vary in terms of their expected diagnostic yields, these may differ according to the operator’s expertise and equipment used. Any method that provides sufficient high-quality material for all the relevant histological and molecular analyses may be adequate provided that they minimise the risk of complications (e.g. bleeding and pneumothorax) for the patient [9].

Minimally invasive techniques can be categorised as follows: (a) flexible bronchoscopy; (b) transthoracic or trans-organ needle biopsy and aspiration; and (c) thoracoscopy. Distant metastatic sites, such as extra-thoracic lymph nodes, liver, skin and bone, can be targeted when the primary tumour is difficult to reach or less suitable for sampling. Table 1 summarises the characteristics, advantages and limitations of each technique.

**Table 1:** Summary of biopsy techniques and sample acquisition methods

Technique	Diagnostic yield (%) [7, 17–22]	Samples	Advantages	Limitations	Common analyses
Samples obtained through a flexible bronchoscope					
Endobronchial biopsy	65–88^a^	• Forceps tissue biopsy sample 2–3 mm • ≥ 5 biopsies (or 2 cryo-biopsies)	• Morphology • Enables PD-L1 TPS, IC and CPS scoring	• Low cellularity	• Histopathologic evaluation • IHC/FISH • Molecular biology • Microbiology (if indicated)
Transbronchial biopsy	65–88^a^				
Bronchial brushings	35–63	• Cytological smears and centrifuge preparations	• Cell pellets • High cellularity^b^	• Not suitable for PD-L1 IC and CPS scoring (only for TPS) • Alcohol fixation	• Smears ○ Cytological evaluation^c^ • FFPE ○ Morphology ○ IHC/FISH ○ Molecular biology • Microbiology (if indicated)
Bronchoalveolar lavage or bronchial washings	~ 42				
Transbronchial EBUS-FNA	75–94				
Samples obtained by transthoracic or trans-organ needle biopsy or aspiration					
Transthoracic or trans-organ fine needle biopsy and aspiration	> 88	• Core biopsy (≥ 2) • Cytological smears and centrifuge preparations	• Morphology • Enables PD-L1 TPS, IC and CPS scoring	• Low cellularity • Alcohol fixation	• FFPE histopathologic evaluation • Smears ○ Cytological evaluation^c^ • IHC/FISH • Molecular biology • Microbiology (if indicated)
Thoracentesis	~ 65	• Cytological smears and centrifuge preparations	• High cellularity • Cell pellets	• Not suitable for PD-L1 IC and CPS scoring • Alcohol fixation	• Smears ○ Cytological evaluation^c^ • FFPE ○ Morphology ○ IHC/FISH ○ Molecular biology • Microbiology (if indicated)
Closed pleural biopsy	38–47	• Small biopsy sample (Abrams, tru-cut, Cope needles)	• Morphology • Enables PD-L1 TPS, IC and CPS scoring	• Low cellularity	• FFPE histopathologic evaluation • IHC/FISH • Molecular biology (if cellularity is sufficient) • Microbiology (if indicated)
Samples obtained by surgical biopsy					
Surgical biopsy of lung parenchyma obtained by thoracoscopy	> 90	• Sample 2–3 cm • Possible sampling of ipsilateral lymph nodes	• High cellularity • Allows PD-L1 TPS, IC and CPS scoring	• Pre-analytics (in case of poor fixation)	• FFPE histopathologic evaluation • IHC/FISH • Molecular biology • Microbiology (if indicated)
Samples obtained by liquid biopsy					
Liquid biopsy	51–75	• 5 mL of whole blood with centrifugation to retrieve 2 mL of plasma	• Non-invasive • Dynamic monitoring	• Sensitivity^d^ • A tissue-based test is required with negative results	• Molecular biology (DNA/RNA)

*CPS* combined positive score, *DNA* deoxyribonucleic acid, *EBUS-FNA* endobronchial ultrasound-guided FNA, *FFPE* formalin-fixed paraffin-embedded, *FNA* fine-needle aspiration, *FISH* fluorescence in situ hybridisation, *IC* immune cell, *IHC* immunohistochemistry, *PD*-*L1* programmed death ligand 1, *RNA* ribonucleic acid, *TPS* tumour proportion score

^a^Up to 94% for cryo-biopsy

^b^For bronchial brushings and transbronchial EBUS-FNA

^c^Papanicolaou [fixed] or Giemsa staining [air dried]

^d^Poor shedding, complex alterations

### Flexible bronchoscopy

Flexible bronchoscopy enables both visual inspection of the major bronchi and sample acquisition and can be performed without anaesthesia. The technique can explore more distal and lateral airway conduits (up to the sixth order bronchi) than rigid bronchoscopy [23]; however, in cases of necrotic or highly vascular tumours, rigid bronchoscopy may be preferred to obtain larger sample yields or improve safety.

Endobronchial biopsy using cupped forceps introduced through the bronchoscope permits sampling of endobronchial tumours from the mucosa down to smooth muscle [24]. Following removal of the device, the sample (2–3 mm) is extricated and fixed immediately. Transbronchial biopsies target the alveolar lung parenchyma beyond the cartilaginous bronchi using either cupped or crocodile-style forceps. At least five endobronchial/transbronchial forceps biopsies should be obtained, and an additional five forceps biopsies or two cryo-biopsies could be considered to maximise tissue volume [9]. In a cryo-biopsy, the lung tissue is rapidly frozen prior to removal and the technique allows for larger samples with reduced artefacts compared with other techniques [17]. Cryo-biopsy has most commonly been used for diagnosing interstitial lung disease [25]; however, studies have shown that it is an efficient sampling technique for lung cancer [17, 25]. Endobronchial/transbronchial cryo-biopsy may increase histological and molecular detection rates of lung tumours when compared with other tissue sampling methods [26, 27], and has been shown to provide a high diagnostic yield (94%) [17]. However, endobronchial/transbronchial cryo-biopsy for lung cancer is not commonly implemented in many countries.

Visualised lesions can be sampled for cytologic evaluation by bronchial brushing, whereby a conical bristle brush is agitated against the pathological lesion, forcing cells into the bristle interstices. This technique offers the advantage of placing smears onto a glass slide for cytological examination and obtaining a suspension of cells after centrifugation for immunohistochemistry (IHC) and molecular testing. The smeared material is fixed immediately by direct immersion in 95% alcohol (e.g. for Papanicolaou staining) or can be air dried (e.g. for Giemsa staining); the choice depends on operator preference and should be discussed with the pathologist. Subsequently, the brush can be cut, immersed in a sterile saline vial and shaken vigorously to dislodge the cells. The fluid sample is sent to the laboratory for centrifugation or filtration; the resulting sample is also suitable for microbiological and/or biochemical assessment.

Bronchoalveolar lavage and bronchial washing are ‘non-lesion-oriented’ sampling techniques. Bronchoalveolar lavage relies on the existence of shed cells within the peripheral lung and airways and involves retrieval of a substantial quantity of saline solution injected into the airways; this allows sampling of secretions and lung parenchyma [28]. This technique is typically used for diagnosing infections in immunocompromised patients and for exploration of interstitial lung disease. Bronchial washing involves aspiration of sterile saline solution near the tips of the bronchoscope. With both techniques, the liquid is centrifuged and the pellets studied if a lung cancer diagnosis is suspected. Bronchoalveolar lavage is currently rarely used for diagnosing lung cancer due to its low sensitivity; however, it may provide key additional information on the local immune reaction in the tumour microenvironment [29].

Transbronchial FNA with or without radial endobronchial ultrasound (EBUS) [7, 15, 30] is usually performed using a 19–20-gauge needle; it has a diagnostic yield of at least 90% in enlarged/bulky lymph nodes [9]. This technique is suitable for diagnosis of central and peripheral lung or node lesions. For cytological assessment, a drop of material can be smeared onto a glass slide using a feathering technique, followed by fixation and staining procedures. The needle is subsequently rinsed and the suspension is centrifuged for further assessment, such as microscopic evaluation, IHC and molecular biology testing.

### Transthoracic or trans-organ needle biopsy and aspiration

Transthoracic core-needle biopsies and fine-needle aspirates are processed in a similar way to specimens obtained through transbronchial needle aspiration (see the previous section). For smears, rapid on-site evaluation (ROSE) of cellularity may help confirm success before termination of the procedure. If a liquid sample is generated, centrifugation or filtration is performed. For core-needle biopsy of a transthoracic or metastatic site, it is recommended to perform at least two needle passes (18–20-gauge needle); three to six CNBs could be considered to maximise tissue volume [9].

Thoracentesis allows cytological examination of pleural effusion. The collected liquid is centrifuged or filtered to obtain a pellet for cytologic assessment, IHC and molecular biology testing, as required.

Closed pleural biopsy allows random sampling of pathologic pleural tissues and several biopsy devices are available to retrieve small samples. With a diagnosis of cancer, IHC and molecular biology tests can be performed, although cellular paucity can be a limitation.

### Surgical biopsy of the lung parenchyma

If the lung target is too small to allow interventional radiology procedures, or if peripheral lesions were missed by endobronchial biopsies, surgical biopsy with video-assisted thoracoscopy (VATS) is indicated; this technique enables larger specimens (2–3 cm). However, since it requires collapse of the affected lung, biopsy of a metastatic lesion may be a safer option.

### Liquid biopsy

‘Liquid biopsy’ is a non-invasive diagnostic technique that includes testing of cell-free DNA (cfDNA) or circulating tumour cells from various body fluids (e.g. blood plasma, pleural fluid, urine or cerebrospinal fluid); of these, testing for cfDNA in blood-derived plasma is the most common [10, 31]. Liquid biopsy testing is associated with limitations and advantages compared with tissue biopsy and should be considered as complementary approaches. For example, liquid biopsy testing generally has lower sensitivity compared with tissue biopsy [10, 31]. However, liquid biopsy can help provide a molecular diagnosis, particularly when the tissue biopsy sample is insufficient or inadequate for molecular analysis. Furthermore, liquid biopsy can be used to monitor response to treatment or by capturing dynamic intra-patient heterogeneity (see Part 2) [10, 31].

### General considerations relating to tissue quality

**Fig. 1:**
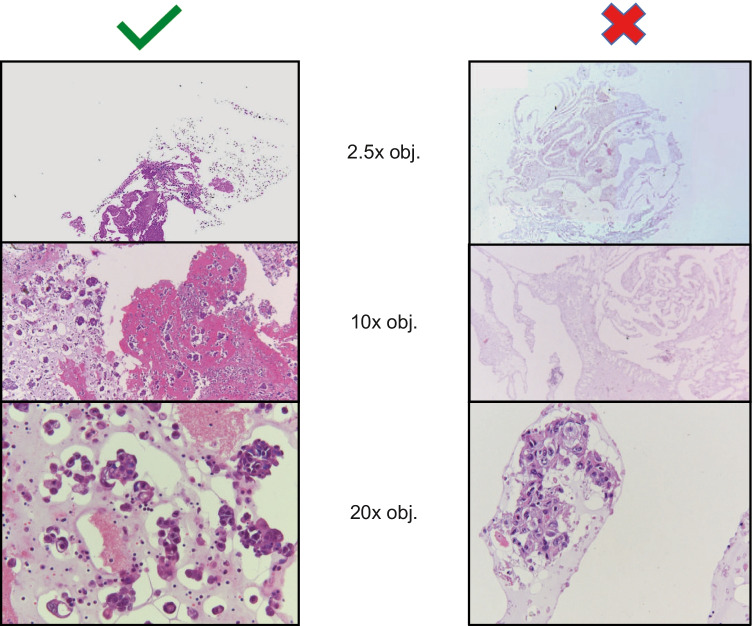
Cytology cell block cellularity can vary from high (left; suitable for molecular analysis) to low (right; too low for DNA/RNA NGS without mutant allele-specific amplification). *DNA *deoxyribonucleic acid, *NGS* next-generation sequencing, *x obj *microscope objective lens magnification, *RNA* ribonucleic acid. Images provided by Erik Thunnissen

Given the limited quantity of tissue available, sample quality is paramount (Fig. 1) [9]. Biopsy tissue quality is expressed as the percentage of neoplastic cells to the total number of nucleated cells in a sample. The percentage of neoplastic cells required for analysis depends on the analytical technique used (e.g. 20–30% for Sanger sequencing or ~ 10% for next-generation sequencing [NGS] [32, 33]). A biomarker-negative sample with a tumour cell content below these thresholds should be determined as inconclusive, requiring further assessment. Consequently, the proportion of cells may also inform the choice of molecular testing technique. In case of low neoplastic cell content, tumour cell enrichment techniques, such as macro-dissection, are recommended for direct sequencing and NGS, highlighting the critical role of the pathologist [34–36]. For NGS, a coverage of 300–500 sequence reads per target is generally adequate for most diagnostics; the exact number of reads required will depend on the neoplastic cell content [37].

If the tissue biopsy is insufficient, or if the complexity of performing the biopsy renders the technique unfeasible, cytological samples or liquid biopsy may provide supplementary diagnostic material [10, 38]. Complementary approaches to optimise the amount of material should therefore be considered. Samples acquired by FNA may have higher tumour cellularity and better sequencing metrics than those acquired by CNB, possibly due to the lower proportion of interfering stromal cells with FNA [39]. Consequently, depending on tumour site and operator experience, pairing biopsy with FNA in the routine diagnostic workflow may lead to a higher success rate in molecular testing [39].

Historically, processing of cytologic and biopsy samples lacked standardisation; to address this, the College of American Pathologists (CAP) developed guideline statements to assist clinicians and pathologists in collecting and processing thoracic small biopsy and cytology tissue samples (Table 2) [40]. Notably, in situ hybridisation technologies, and even NGS, are feasible using cytology preparations [41, 42]. Additionally, ROSE and best practice for tissue processing can help optimise diagnostic yields.

**Table 2:** Guideline statements on the collection and handling of thoracic small biopsy and cytology specimens from the College of American Pathologists in Collaboration with the American College of Chest Physicians, Association for Molecular Pathology, American Society of Cytopathology, American Thoracic Society, Pulmonary Pathology Society, Papanicolaou Society of Cytopathology, Society of Interventional Radiology and Society of Thoracic Radiology [40]

Guideline statement	Strength of recommendation
1. EBUS TBNA may be used, if available, for initial evaluation (diagnosis, staging, identification of recurrence/metastasis) of mediastinal and hilar lymph nodes, as well as centrally located parenchymal lesions visible with endobronchial ultrasound	Strong recommendation
2. When performing EBUS TBNA, 19-, 21- or 22-gauge needles may be used	Recommendation
3. When performing EBUS TBNA, ROSE should be used, if available	Recommendation
4. To achieve optimal diagnostic yield, when performing EBUS TBNA without ROSE, the bronchoscopist should perform at a minimum three and up to five passes, if technically and clinically feasible. When performing with ROSE, clinical judgement should be used to assess the number of passes needed. Additional passes may be required for ancillary studies	Recommendation
5. When performing transthoracic needle procedures, ROSE should be used for adequacy assessment, if available and clinically feasible. If performing CNB without concurrent FNA, touch preparations may be used for adequacy assessment, if available	Strong recommendation for the use of ROSE for adequacy assessment; recommendation for the use of touch preparations without concurrent FNA
6. When performing transthoracic needle procedures, needle size should be determined by the operator and technique. For transthoracic FNAs, needles as small as 25 gauge may be used. For CNBs, needles as small as 20 gauge may be used	Recommendation
7. When performing transthoracic FNA without CNB, the proceduralist should obtain multiple passes, if technically and clinically feasible, and should attempt to collect sufficient material for a tissue block (i.e. cell block, tissue clot)	Recommendation
8. To achieve optimal diagnostic yield when performing transthoracic CNBs, the proceduralist should attempt to obtain a minimum of three core samples, if technically and clinically feasible. Additional samples may be required for ancillary studies	Recommendation
9. If performing bronchoscopy for the investigation of peripheral pulmonary lesions that are difficult to reach with conventional bronchoscopy, image guidance adjuncts may be used, if local expertise and equipment are available	Recommendation
10. When performing transbronchial needle aspirates, ROSE should be used for adequacy assessment, if available. If performing transbronchial forceps biopsies without concurrent transbronchial needle aspirates, touch preparations may be used for adequacy assessment, if available	Recommendation for the use of ROSE for adequacy assessment; expert consensus opinion for the use of touch preparations
11. When collecting pleural fluid for a suspected diagnosis of malignancy, the proceduralist should send as much fluid volume as reasonably attainable for cytologic evaluation and ancillary studies	Expert consensus opinion
12. Cytology specimens (smears, cell blocks, liquid-based cytology) may be used for ancillary studies if supported by adequate validation studies	Strong recommendation
13. CNB specimens collected for ancillary studies should be fixed in 10% neutral-buffered formalin	Recommendation
14. When performing bronchoscopy for the investigation of tuberculosis, EBUS may be used to increase the diagnostic yield of bronchoalveolar lavage and transbronchial biopsy	Recommendation
15. When performing EBUS TBNA for the evaluation of intrathoracic granulomatous lymphadenopathy with the suspicion of tuberculosis, specimens should be collected for cytology, microbiology (mycobacterial smear and culture), and TB-PCR evaluation, if available	Recommendation
16. When collecting pleural fluid for diagnosis of extrapulmonary tuberculosis, specimens should be submitted for microbiology culture studies for mycobacteria using liquid media protocol	Recommendation

Table adapted from Roy-Chowdhuri S, et al. [40] with permission from the College of American Pathologists

*CNB* core-needle biopsy, *EBUS TBNA* endobronchial ultrasound-guided transbronchial needle aspiration, *FNA* fine-needle aspiration, *ROSE* rapid on-site evaluation, *TB-PCR* *Mycobacterium tuberculosis* polymerase chain reaction

## Rapid on-site evaluation (ROSE)

ROSE is recommended by the NCCN Clinical Practice Guidelines in Oncology (NCCN Guidelines®) for NSCLC to ensure transbronchial needle aspirates (TBNAs) or EBUS samples are adequate for molecular testing [16]. It involves performing a rapid stain in the bronchoscopy suite or operating room, with evaluation by a cytopathologist or a cytotechnologist [43], who can confirm the presence of tumour cells and estimate neoplastic cell content [44, 45]. In addition to providing a preliminary diagnosis that may negate the need for further sampling, ROSE helps to ensure sample adequacy and sufficient yield for cancer subtyping and molecular testing [43, 46–48].

ROSE can help achieve a relatively high sampling success rate and reduce the number of required aspirations [45, 46]. However, ROSE can be costly (often with limited reimbursement), time-consuming and require cytopathology expertise that may not always be available [43]. A more cost-effective application may constitute its temporary adoption to establish departmental and multidisciplinary expertise/training [46]. In addition, ROSE can be combined with telecytology to remove the need for an on-site cytopathologist; this method has been shown to be efficient and cost-effective for the diagnosis of lung cancer [43, 49, 50].

## Tissue processing (biopsy samples)

Formalin fixation of surgical samples and those collected using minimally invasive techniques, such as CNB or FNA, is a critical pre-analytical step for biomarker analysis in advanced NSCLC [40]. Formalin fixation and paraffin embedding of tissue samples emerged over 100 years ago as a reliable method for long-term tissue preservation. With the advent of molecular sequencing technologies, biomarker analysis from FFPE sample-derived nucleic acids underpins the diagnosis and management of most solid tumours [16]. Importantly, fixation methods must preserve nucleic acid integrity. The main limitations of formalin fixation in the context of nucleic acid extraction and downstream amplification are fourfold [51]. Firstly, formalin’s use as a fixative is due to its ability to crosslink proteins; however, formalin can also form bridges between proteins and nucleic acids, resulting in potential impurities [52]. Secondly, unbuffered formalin initiates nucleic acid fragmentation via acid-mediated hydrolysis, which may inhibit polymerase chain reaction (PCR) amplification [52]. Thirdly, the quality of DNA in a FFPE sample may auto-degrade slowly over time, although this may not be a frequent practical limitation [53, 54]. A recent study comparing comprehensive NGS on DNA from fresh-frozen versus FFPE samples did not demonstrate any formalin-induced mutational artefacts associated with FFPE samples [55]. Finally, conventional nucleic acid extraction procedures rely on proteinase treatment to reverse formalin-induced crosslinks, which can lead to nucleic acid fragmentation. As the formalin-fixed tissue sample must be suitable for all possible subsequent analyses (e.g. haematoxylin and eosin [H&E] staining, IHC, fluorescence in situ hybridisation [FISH], DNA/RNA sequencing), it is critical to optimise fixation parameters to maximise opportunities for high-quality downstream analysis [56].

RNA is particularly susceptible to degradation and may be influenced by a variety of preanalytical conditions including the length of cold ischaemia, time to fixation and quality and duration of fixation. FFPE samples are an important source of nucleic acids for molecular diagnostics; however, RNA extracted from FFPE samples are often highly fragmented, which has traditionally limited their use [57]. Improvement in NGS technology, which analyses short sequences of nucleic acids, has opened up new uses for RNA extracted from FFPE samples [57], particularly for identifying gene fusions [58]. Coupled with this, recent improvements in techniques to extract, purify and preserve RNA from FFPE samples has enhanced the use of RNA in NGS-based diagnostics [57, 59, 60]. RNA quality should be assessed prior to sequencing to minimise false negative results [61].

Numerous tissue fixation variables influence downstream nucleic acid, protein and morphological analyses. These include sample size, fixative constitution and volume, duration of fixation and cold ischaemia time [55, 62]. In terms of formalin fixation time, to date, no formal, NSCLC-specific recommendations exist. A European expert group recommended an optimal fixation window of 6–48 h [9], based on findings that minimal nucleic acid degradation is observed prior to the 72-h mark. However, the optimal duration of fixation depends on the quantity of tissue, and 6 h may not be sufficient for larger specimens. If formalin fixation is incomplete, subsequent alcohol processing can alter the antigenicity of certain epitopes. For small NSCLC samples, 6–24-h fixation time (where feasible) is a conservative approach to minimise nucleic acid degradation. In many cases, practical constraints, such as the availability of laboratory resources at weekends, drive institutional standards towards a 48-h maximum.

The CAP/American Society of Clinical Oncology (ASCO) guidelines for the collection and handling of thoracic small biopsy and cytology samples recommend 10% neutral-buffered formalin as a fixative for CNB [40]. Non-conventional fixatives, such as Bouin solution and other acid- or heavy metal-containing solutions, are not recommended because of the potential for impurities or degradation that would compromise downstream PCR amplification. In bone biopsies, decalcification with harsh acids leads to nucleic acid degradation and should be avoided [63]. Ethylenediaminetetraacetic acid (EDTA) is more conducive to preservation of nucleic acids but requires longer fixation. Notably, many laboratories automatically send bone biopsies for decalcification, but this may not always be required. Bone metastases of NSCLC are often osteolytic; therefore, case-by-case assessment by the pathologist is recommended. Manual palpation of each sample is often amenable to teasing off soft tissues for contiguous submission. If necessary, decalcification should be performed as last resort, and EDTA is recommended [59].

**Fig. 2:**
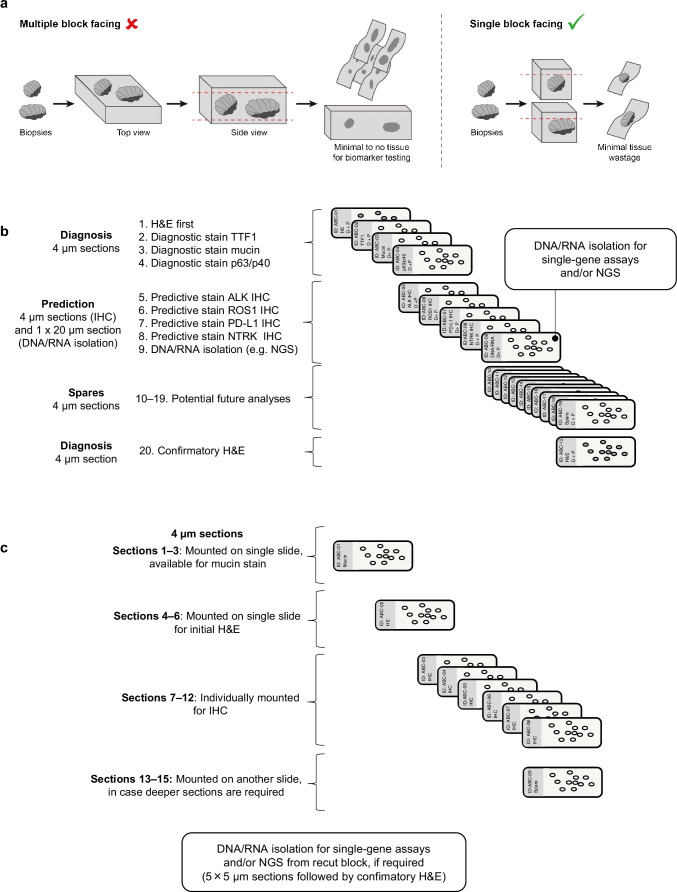
Approaches to avoid tissue wastage. ‘One biopsy per block’ approach (**a**) and tissue-sparing cutting protocols: Diagnose and predict (D + P) protocol, as used by author E. Thunnissen in Amsterdam (**b**), and an alternative approach, as used by author K. Kerr in Aberdeen (**c**). *ALK *anaplastic lymphoma kinase, *H&E* haematoxylin and eosin, *IHC* immunohistochemistry, *NGS* next-generation sequencing, *NTRK *neurotrophic tyrosine receptor kinase, *PD-L1* programmed cell death ligand 1, *ROS1* ROS proto-oncogene 1, *TTF1* thyroid transcription factor-1

In addition to optimal fixation methods, tissue sparing techniques are important in the pathological work-up (Table 3) [64]. Following fixation, tissue sparing strategies can be implemented, including a ‘one biopsy per block’ approach to embedding (Fig. 2a) and small biopsy block cutting protocols (Fig. 2b; see section ‘Tumour diagnosis and subtyping’). The ‘one biopsy per block’ approach involves embedding each individual formalin-fixed tissue core or aspirate into a separate paraffin-embedded cassette, which avoids tissue loss associated with resurfacing blocks with multiple cores embedded at slightly different planes. Although this approach is associated with an increased consumable cost, overall cost savings compared with the potential for tissue rebiopsy may be considerable. Benefits to the patient include a reduced likelihood of incomplete/inadequate biomarker testing and a reduced chance for rebiopsy.

**Table 3:** A summary of evidence-based recommendations around key aspects of small specimen biomarker testing in NSCLC

Key opinions and recommendations^a^
***Biopsy techniques*** • Biopsy technique options are dependent on the disease site [16] and include: ○ Core-needle biopsy (transthoracic or metastatic site): 2–6 biopsies with an 18–20-gauge needle [9] ○ Bronchoscopic forceps biopsy: at least five endobronchial/transbronchial forceps biopsies should be obtained and an additional five forceps biopsies or two cryo-biopsies could be considered, where feasible [9] ○ Transbronchial FNA with or without radial EBUS: at least four needle aspiration passes per target lesion are recommended with a 21- or 22-gauge needle [9] ○ Endoscopic ultrasound with bronchoscope-guided FNA is usually employed when EBUS-transbronchial FNA is not feasible or when excessive cough or secretions necessitate a switch to the oesophageal route ○ Pulmonary samples such as effusions and exfoliative specimens are a source of supplementary patient material [15] • Tissue quality/quantity considerations: ○ For NGS, a minimum of ~ 10% of a sample for genetic alteration testing should be made up of neoplastic cells to minimise false-negative results [32, 33]; if necessary, apply macro-microdissection to enrich tumour sample. A coverage of 300–500 sequence reads per target is generally adequate for most diagnostics; the exact number of reads required will depend on the neoplastic cell content [37] ○ Complementary approaches to optimise the amount of material should be considered (e.g. PET-CT) [65] ○ Tumour cell enrichment techniques such as macro-dissection are recommended for direct sequencing and NGS [34] ○ If the tissue biopsy is insufficient or impractical, cytological samples and liquid biopsy might be appropriate [10] ○ Combining biopsy with FNA in the routine diagnostic workflow may lead to a higher success rate in molecular testing [39]
***ROSE*** • ROSE is recommended by the NCCN Guidelines® for NSCLC to ensure TBNAs or EBUS specimens are adequate for molecular testing [16] ○ A cost-effective application of ROSE may be temporary adoption to establish departmental and multidisciplinary expertise/training [46]
***Tissue processing (biopsy samples)*** • Optimise tissue processing to maximise DNA quality (e.g. fix in 10% neutral-buffered formalin for 6–48 h; avoid unbuffered formalin or fixatives containing acids or heavy metals; perform decalcification only as last resort and using EDTA) [9, 40, 59, 62, 63] • Separate tissue fragments into individual blocks (i.e. ‘one sample per block’ approach; this reduces wastage associated with facing that would be required to achieve a plane including multiple tissue fragments) [64]
***Cytological sample processing*** • Judicious collection and utilisation of cytopathology samples (e.g. FFPE blocks, smears, touch preparations) can compensate for insufficient tumour tissue and, in some cases, may serve as the primary material [8, 66] • Implement best practice algorithms to maximise neoplastic cells in cytological samples (e.g. ROSE) [15, 48] • Consider the use of body fluids (e.g. pleural or pericardial fluid) as a specimen modality for molecular diagnostics [67, 68]. Approximately 10–15 mL should be sent for biochemical analysis and cell culture, with the remaining fluid volume sent to cytology for processing
***Morphological analysis*** • Consider use of a ‘Diagnose and Predict’ protocol (see Fig. 2) • Cut extra sections at the first cutting session (i.e. small biopsy block cutting protocol) [9] • Minimise the frequency of recutting blocks [9] • Foster a proactive attitude in the clinic, whereby clinicians anticipate downstream testing requirements • Consider the use of paired morphological and cytological samples to maximise the amount of cytological sample available for molecular diagnosis

^a^Where no guidelines or literature explicitly describe best practice, recommendations for best practice are reported according to the experience of the author group

*DNA* deoxyribonucleic acid, *EBUS* endobronchial ultrasound, *EDTA* ethylenediaminetetraacetic acid, *FFPE* formalin-fixed paraffin-embedded, *FNA* fine-needle aspiration, *NGS* next-generation sequencing, *PET-CT* positron emission tomography-computed tomography, *ROSE* rapid on-site evaluation, *TBNA* transbronchial needle aspirate

## Processing of cytological material

Judicious collection and utilisation of cytopathology samples are important in compensating for insufficient tumour tissue and may serve as the primary material for molecular testing. In many centres, the only samples available for all diagnostic steps are cytological specimens. Cytopathology samples typically refer to cellular material devoid of tissue stroma, and exfoliated or disaggregated cellular material. Collection techniques include EBUS-guided FNA, endobronchial cytology, bronchoalveolar lavage, pleural effusions and FNA from distant metastases [9, 66]. Cytological material is useful in establishing a morphological diagnosis and provides a source of nucleic acids for molecular testing [9, 69]. The procedures have different strengths and limitations. For example, smears from endobronchial brushing or EBUS-guided FNA may contain necrotic material as well as neoplastic elements. EBUS samples may also contain non-neoplastic tissue from lymph nodes and the amount of material collected may be limited. In contrast, pleural effusions usually contain large amounts of neoplastic cells, but frequently also include a substantial portion of admixed reactive mesothelial cells and/or inflammatory cells. ROSE can also improve the quality and success rates of molecular testing with EBUS-FNAs [15, 48].

An additional consideration associated with cytological specimens is adequate morphological classification. In patients with sufficient tissue for morphological diagnosis, the entire cytology specimen can be utilised for molecular testing. In contrast, if tissue is inadequate or unavailable, cytological material must be used for both diagnosis and molecular testing. Thus, samples from each patient must be considered individually. In any case, material should be maximised to attain as much information as possible and provide clinicians with a comprehensive morphological/molecular report to enable treatment selection [16].

Cytopathology samples are classified as either FFPE cell blocks [70, 71] or non-FFPE cytopathology (NFC) samples. NFC samples, such as smears and touch preparations, are fixed in ethanol or methanol. In the absence of formalin fixation, these samples typically contain higher-quality nucleic acids than FFPE samples and are useful for molecular testing [8]. For example, to isolate 10 ng of nucleic acids (typical minimum input for most NGS platforms) [72], approximately 3–fourfold more cells are required from a FFPE sample than from ethanol-fixed material. The required cell number can usually be obtained by scraping cells from a smear. It is beneficial if the pathologist indicates the areas containing the highest proportion of neoplastic cells on the slide [9] because it helps to avoid contamination of the material for nucleic acid extraction by non-neoplastic elements. Furthermore, if referred for molecular analysis prior to cover-slipping, the time to nucleic acid extraction can be reduced to 1 day.

The suitability of NFC samples for molecular biomarker testing was highlighted in the most recent CAP/IASLC/Association for Molecular Pathology (AMP) guidelines [73]. The utility of FNA smears for broad-panel (46-gene) NGS testing has also been reported in various publications. For example, Kanagal-Shamanna and colleagues found that DNA of sufficient quantity and quality for NGS was extracted from 48% of FNA smears (*n* = 33) [72]. In the authors’ experience, most molecular pathology laboratories accept such cytopathology samples. However, most molecular testing using cytological material is now performed with cell blocks rather than smears.

Molecular testing on FFPE cell blocks can be as sensitive and specific as that on tissue specimens [71]. FFPE cell blocks are prepared by centrifugation of a cytological sample (e.g. pleural effusion, bronchial washing or needle rinse following FNA puncture). The pellet is then formalin fixed and paraffin embedded [70]. Specialised block preparation and cutting techniques can improve the quality and quantity of tumour cells [70], although the experience of the technician is crucial. Cell blocks have been reported to provide adequate samples for NGS in around 87–94% of cases [74]. One limitation associated with the use of cell blocks is lack of standardisation in processing techniques. Several commercial cell block production techniques are available, with one common method employing expired donor-derived plasma and thrombin to congeal the centrifuged cell pellet [15]. Utilisation of donor plasma can theoretically contribute analytical error via donor-derived DNA [15]. With regards to method validation, as the fixation protocols for FFPE blocks and NFC samples differ, laboratories must take steps to ensure that the reliability of results from DNA extracted from both sample types is the same.

Body fluids represent an important, yet challenging, specimen modality for molecular diagnostics [67, 68]. Tumour cells may be difficult to distinguish in fluid, making enrichment difficult. Relevant body fluids for molecular analysis of advanced NSCLC include pleural and pericardial fluid [68] and bronchoalveolar lavage. It is therefore important that this material is not inadvertently discarded during the diagnostic or therapeutic procedure, requiring careful communication with the multidisciplinary team and inclusion in relevant procedural protocols. Approximately 10–15 mL should be sent for biochemical analysis and cell culture (if appropriate), with the remaining fluid volume sent to cytology for processing. Downstream molecular analysis of such samples should be conducted using high-sensitivity sequencing platforms and any negative results must carry an appropriate disclaimer owing to the higher probability of false-negative results when tumour content is low.

Overall, cytology is an excellent source of material for molecular testing and provides an equivalent (or superior) alternative to tissue [8, 66]. Regardless of the source of tumour cells, the overarching principle is that care should be taken to ensure that the sample submitted for molecular testing contains sufficient neoplastic cells and that their relative proportion is known. It is the responsibility of the pathologist to ensure that nucleic acids extracted from the cytological sample are derived from an adequate proportion of tumour cells.

There are important administrative and legal considerations when using cytopathological samples for molecular diagnostic analysis. It is recommended that laboratories define policies for partial or complete use of cytopathology slides, as well as for conditions around procuring a slide specifically for molecular testing. Using diagnostic cytopathology material for molecular testing can destroy evidence for morphological diagnosis. Some laboratories will digitally scan the slide before sending it for molecular testing while others will find it more practical to pursue FFPE cell blocks, which enables archiving and storage of remaining sample.

## Tumour diagnosis and subtyping

Recommendations on diagnostic approaches are well established in the 2021 WHO Thoracic Classification of Tumours [75]. In brief, assessment of the tumour involves several questions, including the following: Is this carcinoma, as opposed to another broad group of malignant tumours? If so, is it likely to be primary lung cancer? Is the tumour small cell carcinoma? Pathologists need to be alert to the possibility of metastatic disease to the thorax but ought to be judicious when determining how vigorously to pursue an alternative source for an obvious adenocarcinoma (based on morphology, clinical history and clinical opinion). Valuable tissue may be wasted on unnecessary IHC in pursuit of uncommon or unlikely alternatives, and this can result in insufficient material being available for subsequent biomarker testing [76]. In up to 70% of small biopsy or cytology samples, morphology alone is sufficient to achieve a diagnosis more specific than non-small-cell carcinoma not otherwise specified (NSCC-NOS) [77, 78]. IHC should only be used in the remaining cases where the best morphological diagnosis is NSCC-NOS, in order to predict the subtype [79–82]. The use of IHC should be limited and only two markers are required, TTF1 and p40 (or p63); double staining IHC allows four IHC markers on two slides. Most cases need no more than this, which leaves more pre-cut sections for biomarker IHC (e.g. ALK, programmed death-ligand 1 [PD-L1]). Guidelines recommend that routine biomarker testing (excluding PD-L1) is largely confined to cases diagnosed as definite/probable adenocarcinoma or where this subtype cannot be reasonably excluded. Strategies may be put in place in the laboratory to maximise utilisation of meagre tumour samples, and examples of such strategies are detailed in the following paragraphs.

At Amsterdam UMC (author E. Thunnissen), the clinician assigns the request for a predictive analysis on the test form as ‘diagnosis + prediction’ (coded as D + P) [66]. In the laboratory, the D + P code is transmitted to the cassette of the paraffin block. Laboratory technicians are aware that the D + P code requires a specific cutting process: (a) new/clean knife on the microtome; (b) a superficial cut of the FFPE embedded biopsy; (c) a few ribbons of paraffin sections starting at 4 μm, one ribbon of 20-μm and a final 4-μm section (Fig. 2b); (d) orderly transfer of approximately 20 × 4-μm sections and the final 4-μm section to a microscope glass slide (one section per slide). The remainder of the ribbons are stored on the shelf for three working days. Subsequently, the slides containing the ‘first’ and ‘last’ 4-μm sections are H&E-stained.

Pathologists use the two H&E-stained slides for morphological diagnosis. If needed (e.g. if the carcinoma is poorly differentiated), the pathologist may request additional diagnostic stains, such as TTF-1 for adenocarcinoma, p63/p40 for squamous carcinoma or mucin stain [83]; predictive test(s) can also be requested (e.g. NGS to identify oncogenic alterations). The 20-μm section can be used for DNA/RNA isolation and the remaining 4-μm sections are used for IHC (e.g. PD-L1) and/or FISH. Of note, these extra sections are derived from tissue between the first and last H&E-stained slides, certifying the presence of tumour cells and allowing for an estimate of tumour cell fraction, while the clean knife helps to avoid contamination. In addition, since all the cutting is done immediately, no material is lost. Replacing and resurfacing the block would lead to unnecessary material loss.

More FFPE sections are cut using the D + P process than conventional cutting approaches (Amsterdam UMC experience). However, in terms of efficiency, no deeper cuts are needed with the D + P process, as would be required by conventional cutting protocols in many laboratories. Based on experience with the D + P protocol at Amsterdam UMC, in a 6-month period, the extra sections were used for IHC in 81% of cases and for predictive DNA testing in 52% of the cases. Thus, in 81% of cases, a recut of the FFPE block was avoided using the D + P approach. In economic terms, cutting the extra slides while the block is already in the microtome has a minor impact on technician time compared with the additional time required for recutting using the conventional approach (i.e. locating the FFPE block, placing it in the microtome and adjusting it to the surface of the knife). The process becomes even more inefficient when different technicians are responsible for cutting sections for IHC and DNA testing, because the handling time is doubled, which, in turn, increases the risk that the end of the small sample will be reached. The D + P process also speeds up the turnaround time if additional diagnostic and/or predictive tests are required, as the cutting part will have already been performed. Many laboratories are now adopting the D + P process [66] for handling of NSCLC samples.

In Aberdeen (author K. Kerr), all small biopsy samples of thoracic origin that arrive in the laboratory are handled as follows: at the first cutting of the block, 4-μm sections with any tissue are mounted sequentially on slides (Fig. 2c): sections 1–3 on one slide, 4–6 on a second slide, 7–12 individually mounted on slides for IHC, and 13–15 on another slide (a similar process is used by author H. Moch in Zurich but with more sections). Sections 1–3 are those taken to ‘face-up’ the block (often discarded in traditional sample processing protocols). Sections 4–6 are used for initial H&E staining while sections 1–3 are sometimes used for a mucin stain, and sections 13–15 are used if deeper sections are required. These extra sections and the IHC slides are all available more quickly through prospective, reflex sectioning of the blocks. If DNA/RNA extraction is required, the block is recut using a dedicated, molecularly sterile microtome in a different room from that used for routine section cutting. These steps have ensured a rapid turnaround time for diagnosis (including required IHC) and have minimised cases where tumour material is consumed by the early diagnostic steps. Careful marking of five 5-μm-thick blank sections for DNA/RNA extraction in every case maximises tumour content by microdissection; failure rates using targeted NGS range from 1 to 5% and previous problems with contamination have been eliminated.

## Conclusions

As most patients with lung cancer are diagnosed at an advanced, inoperable stage, small samples from endobronchial/transthoracic biopsies are often the only material available and are provided as tissue or cytological specimens. The appropriate qualified decision regarding optimal treatment selection requires several key steps, including morphological subtyping and biomarker testing, with reflex testing being the best approach to save tissue and time. Multiple best practices (Table 3) can be employed to optimise available tissue and help ensure all patients receive high-quality biomarker testing and the opportunity to benefit from targeted therapy if their tumours harbour appropriate driver mutations.

## Data Availability

Data sharing is not applicable. No new data were created or analysed in this article.
